# Expression Analysis of an R3-Type MYB Transcription Factor *CPC-LIKE MYB4* (*TRICHOMELESS2*) and *CPL4*-Related Transcripts in *Arabidopsis*

**DOI:** 10.3390/ijms13033478

**Published:** 2012-03-13

**Authors:** Rumi Tominaga-Wada, Yuka Nukumizu

**Affiliations:** Interdisciplinary Research Organization, University of Miyazaki, 1-1, Gakuen Kibanadai-Nishi, Miyazaki 889-2192, Japan; E-Mail: y-nukumizu@cc.miyazaki-u.ac.jp

**Keywords:** *Arabidopsis*, epidermal cell differentiation, MYB, transcription factor, trichome

## Abstract

The *CAPRICE* (*CPC*)-like MYB gene family encodes R3-type MYB transcription factors in *Arabidopsis*. There are six additional *CPC*-like MYB sequences in the *Arabidopsis* genome, including *TRYPTICHON* (*TRY*), *ENHANCER OF TRY AND CPC1* and *2* (*ETC1* and *ETC2*), *ENHANCER OF TRY AND CPC3/CPC-LIKE MYB3* (*ETC3/CPL3*), and *TRICHOMELESS1* and *2* (*TCL1* and *TCL2*). We independently identified *CPC-LIKE MYB4* (*CPL4*), which was found to be identical to *TCL2*. RT-PCR analysis showed that *CPL4* is strongly expressed in shoots, including true leaves, but not in roots. Promoter-GUS analyses indicated that *CPL4* is specifically expressed in leaf blades. Although *CPC* expression was repressed in 35S::*ETC1*, 35S::*ETC2* and 35S::*CPL3* backgrounds, *CPL4* expression was not affected by *ETC1*, *ETC2* or *CPL3* over-expression. Notably, several chimeric transcripts may result from inter-genic alternative splicing of *CPL4* and *ETC2*, two tandemly repeated genes on chromosome II. At least two chimeric transcripts named *CPL4-α* and *CPL4-β* are expected to encode complete CPC-like MYB proteins.

## 1. Introduction

Epidermal cell differentiation in *Arabidopsis*, including root-hair and trichome cell formation, has been used as a model system to analyze plant cell fate determination. Several regulatory factors are known to be involved in epidermal cell differentiation events. The *glabra 2* (*gl2*) and *werewolf* (*wer*) mutants induce an increased number of root-hair cells [1,2]. The *GL2* gene encodes a homeodomain leucine-zipper protein, and the *WER* gene encodes an R2R3-type MYB transcription factor that activates *GL2* expression [1–4]. The *GL1* and *MYB23* genes encode R2R3-type MYB genes that are closely related to *WER* and are also involved in epidermal cell fate determination in *Arabidopsis*. The *GL1* gene promotes trichome formation, and the *gl1* mutant phenotype shows a reduced number of leaf trichomes [5]. *GL1* is expressed in developing trichomes [6]. Constitutive expression of the *MYB23* gene under the control of the CaMV 35S promoter induces ectopic trichome formation [7]. *MYB23* and *WER* are preferentially expressed in non-hair cells in *Arabidopsis* roots [8,9]. *GLABRA3* (*GL3*) and *ENHANCER OF GLABRA3* (*EGL3*) encode basic helix-loop-helix (bHLH) transcription factors that affect non-hair cell differentiation in a redundant manner [10]. There are two other bHLH genes, *AtMYC1* [11] and *TRANSPARENT TESTA8* (*TT8*) [12], that are in the same gene family as *GL3* and *EGL3* [13]. The *TRANSPARENT TESTA GLABRA1* (*TTG1*) gene encodes a WD40-repeat protein that regulates non-hair cell formation and trichome differentiation [14,15]. Using a yeast two-hybrid system, GL3 and EGL3 were shown to interact with WER [10] and with a WD40 protein (TTG1) [16–18].

The *CAPRICE* (*CPC*) gene encodes an R3-type small MYB protein, and the *cpc* mutation strongly reduces the formation of root hairs [19]. Previously, we reported that the CPC protein moves from non-hair cells to root-hair cells and represses *GL2* expression [20,21]. In addition, we proposed a model in which the *CPC* gene could have arisen by evolution from the *WER* gene [22]. A protein complex including WER, GL3/EGL3 and TTG1 induces *GL2* expression [2,3,14,19,23]. The CPC protein disrupts this protein complex by competitively binding with WER, leading to repression of *GL2* expression [24,25]. *Arabidopsis* has several additional *CPC*-like MYB sequences in its genome, including *TRYPTICHON* (*TRY*), *ENHANCER OF TRY AND CPC1* and *2* (*ETC1* and *ETC2*), *ENHANCER OF TRY AND CPC3/CPC-LIKE MYB3* (*ETC3/CPL3*), and *TRICHOMELESS1* and *2* (*TCL1* and *TCL2*) [26–33]. Trichome cluster formation on leaves of the *try* mutant indicates that the TRY protein has a regulatory role in trichome differentiation [26,34]. *ETC1* and *ETC2* have been identified and their function with *CPC* and *TRY* genetically examined [27–29]. *TCL1* and *TCL2* negatively regulate trichome formation on the inflorescence stems and pedicels [32,33]. We have recently identified a seventh *CPC*-like MYB gene between At2g30430 and *ETC2* (At2g30420) independently of Gan *et al.* [33], and have named it *CPC-LIKE MYB4* (*CPL4*). In this paper, we examine the expression of the *CPL4* gene in *Arabidopsis*. Notably, between *CPL4* and *ETC2*, there were several chimeric transcripts generated through alternative splicing. Around 14% of the protein coding genes of *Arabidopsis* are annotated as producing multiple transcript variants through alternative splicing [35]. Our study proposes that inter-genic alterative splicing also characterizes the *CPC*-like MYB gene family.

## 2. Results and Discussion

### 2.1. *CPC-LIKE MYB4* (*CPL4*) Gene in *Arabidopsis*

**Figure 1:**
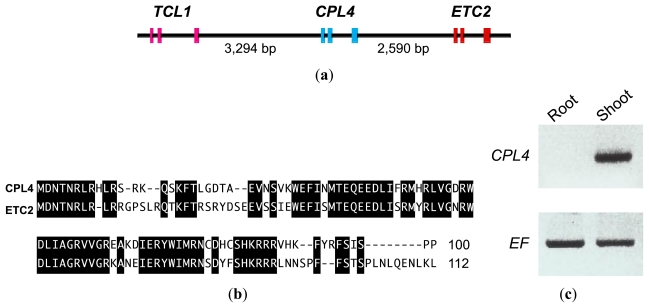
Genomic structure, amino acid sequence and expression of the *CPL4* gene. (**a**) Structure of tandemly arranged genes, *ETC2*, *CPL4* and *TCL1*, in the genome. The exons are represented by pink (*ETC2*), blue (*CPL4*) and red (*TCL1*). The lengths of DNA sequences between genes are shown in base pairs; (**b**) Amino acid sequence comparison of CPL4 and ETC2 proteins generated using Genetyx ver.16.0.2 software [36]. Identical amino acids are shaded in black; (**c**) Expression of *CPL4* in shoots of *Arabidopsis* seedlings. Tissues from *Arabidopsis* roots or shoots were collected, RNA was isolated, and RT-PCR was performed to check for the expression of *CPL4*. The PCR product size using primer pair (RT341/RT342) was 241 bp. The expression of *Elongation Factor1α* (*EF*) was used as a control.

*CPC* encodes an R3-type MYB transcription factor and promotes root-hair cell differentiation [19]. A search of the *Arabidopsis* genome sequence revealed six MYB gene sequences with high homology to *CPC: TRY*, *ETC1*, *ETC2*, *CPL3/ETC3* and *TCL1* [26–32]. In addition to these six *CPC-*like MYB genes, we independently identified the *CPC-LIKE MYB4* (*CPL4*) gene in the *Arabidopsis* genome. The *CPL4* gene encodes a *CPC-LIKE R3-type MYB* sequence and is situated between *TCL1* (At2g30432) and *ETC2* (At2g30420) (Figure 1a). Amino acid sequence alignment showed that these two tandemly repeated genes, *CPL4* and *ETC2*, share high homology (Figure 1a,b). *CPL4* was identical with *TRICHOMELESS2* (*TCL2*) [33]. The CPL4 protein shares 80% amino acid homology with TCL1, 70% with TRY, 68% with ETC2, 49% with CPL3/ETC3, 48% with CPC and 47% with ETC2. As expected from the amino acid sequence of CPL4, overexpression of *CPL4* resulted in a glabrous phenotype similar to *CPC*, *TRY*, *ETC1*, *ETC2*, *CPL3/ETC3* or *TCL1* overexpressors [33].

*CPL4* expression was examined in *Arabidopsis* roots and shoots using semi-quantitative RT-PCR analysis (Figure 1c). *CPL4* was strongly expressed in shoots (including a few small true leaves). We did not detect *CPL4* expression in *Arabidopsis* seedling roots (Figure 1c). Through this assay, we confirmed the expression of a seventh *CPC*-like MYB gene that had not been previously recognized.

### 2.2. Promoter-GUS Analysis

**Figure 2:**
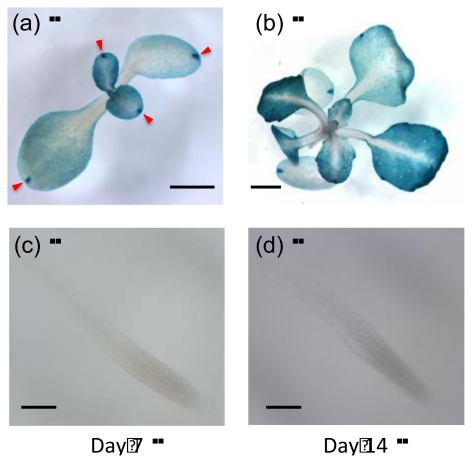
Expression of *CPL4p::GUS* in *Arabidopsis* leaves and roots. (**a**) *CPL4p::GUS* expression in cotyledons and true leaves of 7-day-old seedlings. Arrowheads indicate hydathodes; (**b**) *CPL4p::GUS* expression in two-week-old rosette leaves; (**c**) *CPL4p::GUS* expression in roots of 7-day-old seedlings; (**d**) *CPL4p::GUS* expression in roots of two-week-old plants. Scale bars, 1 mm ([**a**] and [**b**]), 100 μm ([**c**] and [**d**]).

To analyze *CPL4* expression at the tissue level, we made *CPL4* promoter-GUS fusions. *CPL4p::GUS* was expressed in young true leaves and cotyledons of 7-day-old seedlings (Figure 2a). *CPL4p::GUS* expression was also observed in two-week-old rosette leaves (Figure 2b). Previously, we showed that the expression patterns of *CPC*-like MYB genes could be roughly classified into two groups [31]. *CPCp::GUS*, *TRYp::GUS* and *ETC1p::GUS* are expressed mainly in roots and trichomes, and *ETC2p::GUS* and *CPL3p::GUS* are expressed in young leaves and mainly in guard cells [31]. Thus, GUS expression by the *CPC*-like MYB family is found in tissues throughout the entire plant body. We did not detect *CPL4p::GUS* expression in trichomes. Unexpected strong *CPL4p::GUS* expression was observed in hydathodes (Figure 2a). Consistent with the results of RT-PCR (Figure 1b), we did not detect *CPL4p::GUS* expression in 7-day- and two-week-old *Arabidopsis* roots (Figure 2c,d).

### 2.3. *CPL4* Expression in *35S::ETC1*, *35S::ETC2* and *35S::CPL3/ETC3*

**Figure 3:**
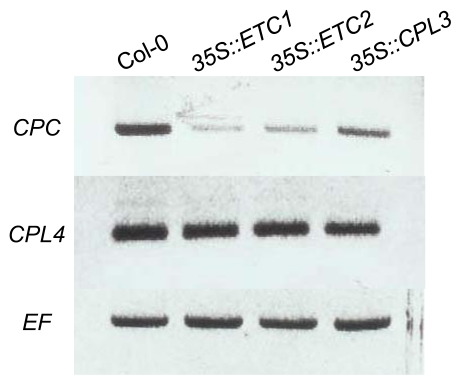
Semi-quantitative RT-PCR analyses of *CPL4* expression in *Arabidopsis*. Tissues from 12-day-old rosette leaves of *Arabidopsis* seedlings were collected, RNA was isolated, and RT-PCR was performed to investigate the expression of *CPC* and *CPL4* in *35S::ETC1*, *35S::ETC2* or *35S::CPL3* overexpressing backgrounds. The expression of *EF* was used as a control.

In addition to the spatial expression patterns, *CPC* expression was regulated by the CPC MYB protein itself as shown in *35S::CPC* transgenic lines [24]. The mechanism for this repression may be by negative feedback, thereby contributing to the root-hair and non-hair cell differences. Because CPC protein shares high amino acid sequence homology with the other CPC-like MYBs, we checked the effect of ETC1, ETC2 or CPL3 overexpression on *CPC* expression (Figure 3). Semi-quantitative RT-PCR analyses showed that *CPC* expression was clearly repressed in the *35S::ETC1*, *35S::ETC2* and *35S::CPL3* backgrounds (Figure 3). This result suggests the existence of a similar feedback loop resulting in the proper trichome distribution on leaves. To compare the regulation of gene expression, we also performed RT-PCR analyses using *CPL4*-specific primers. In plants harboring *35S::ETC1*, *35S::ETC2* and *35S::CPL3*, *CPL4* was expressed at almost the same level as wild-type (Col-0) (Figure 3). These results suggest that *CPL4* is not involved in a feedback regulatory cascade controlled by *CPC*-like MYBs. The *CPC*-like MYB family members are thought to have evolved by gene duplication [31] and have diverged to different regulatory functions in the course of evolution. The *CPC*-like genes retain some functional redundancy that may represent intermediate stages of regulatory specification [37,38]. Thus, *CPL4* may have acquired specific functions different from that of *CPC*.

### 2.4. *CPL4*-Related Gene

#### 2.4.1. *CPL4*-Related Gene Expression

**Figure 4:**
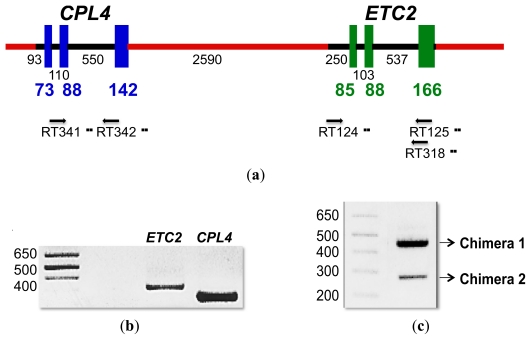
Existence of *CPL4*-related chimeric transcripts. (**a**) Diagram illustrating the *CPL4* and *ETC2* genes located on *Arabidopsis* chromosome II. The blue boxes represent *CPL4* exons. The green boxes represent *ETC2* exons. The black lines represent introns and UTR. The red lines represent intergenic DNA. The lengths of DNA sequences are noted in base pairs. The positions of the PCR primers used in the RT-PCR are indicated with arrows; (**b**) Expression of *ETC2* and *CPL4* transcripts in *Arabidopsis* leaves. RNA was isolated from 12-day-old rosette leaves, and RT-PCR was performed to examine the expression of *ETC2* and *CPL4* transcripts. The size of the marker in base pairs is shown on the left; (**c**) Expression of *ETC2* and *CPL4* chimeric transcripts in *Arabidopsis* leaves. RNA was isolated from 12-day-old rosette leaves, and RT-PCR was used to examine the expression of chimeric transcripts. The size of the marker in base pairs is shown on the left.

*CPL4* is 2590 bp upstream of the other *CPC*-like MYB gene, *ETC2*, on chromosome II and lies in a head-to-tail orientation (Figures 1a and 4a). Both genes are composed of three exons (Figures 1a and 4a). As shown in Figure 1b, *CPL4* shares high amino acid sequence homology with *ETC2*. Therefore, the forward primer beginning with the predicted start codon of *ETC2* can also act as the forward primer beginning with the start codon of *CPL4*. Therefore, it was necessary to carefully design primer pairs specific for *ETC2* and *CPL4*. Gene-specific RT-PCR was performed for *ETC2* and *CPL4* using rosette leaves from 12-day-old *Arabidopsis* seedlings. Both primer pairs designed for *ETC2* (RT124/RT125) and for *CPL4* (RT341/RT342) amplified specific, single gene products of the expected sizes (320 bp and 240 bp) (Figure 4b). Using the *CPL4*-specific forward primer (RT341) and *ETC2*-specific reverse primer (RT125) as a primer pair, two DNA fragments named *CPL4*-related Chimera 1 and Chimera 2 were amplified (Figure 4c). An abundantly expressed band (Chimera 1) was estimated to be approximately 450 bp, and a less expressed band (Chimera 2) was approximately 280 bp in size (Figure 4c). To confirm the existence of *CPL4*-related chimeric gene expression, we repeated the RT-PCR experiment using new RNA samples and another primer pair (RT341/RT318). As a result, the existence of Chimera 1 and 2 cDNA products was confirmed.

#### 2.4.2. *CPL4*-Related Chimera Sequences

**Figure 5:**
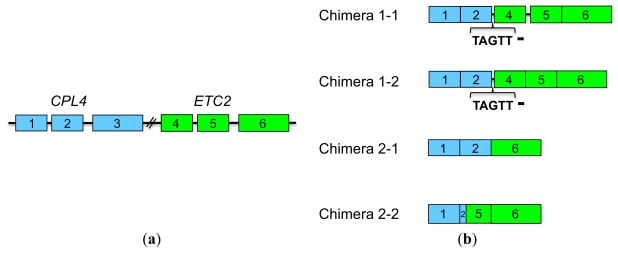
Gene structure of *CPL4*-related chimeric transcripts. (**a**) Structure of *CPL4* and *ETC2* genes. The blue boxes 1, 2 and 3 represent *CPL4* exons. The green boxes 4, 5 and 6 represent *ETC2* exons. The black lines represent introns; (**b**) Structure of *CPL4*-related chimeric transcripts, Chimera 1-1, 1-2, 2-1 and 2-2, produced from *CPL4* and *ETC2* by intergenic alternative splicing. The blue boxes 1, 2 and 3 represent *CPL4* exons. The green boxes 4, 5 and 6 represent *ETC2* exons. The black lines represent introns. The linker sequences have been inserted between *CPL4* and *ETC2* in Chimera 1-1 and Chimera 1-2.

**Figure 6:**
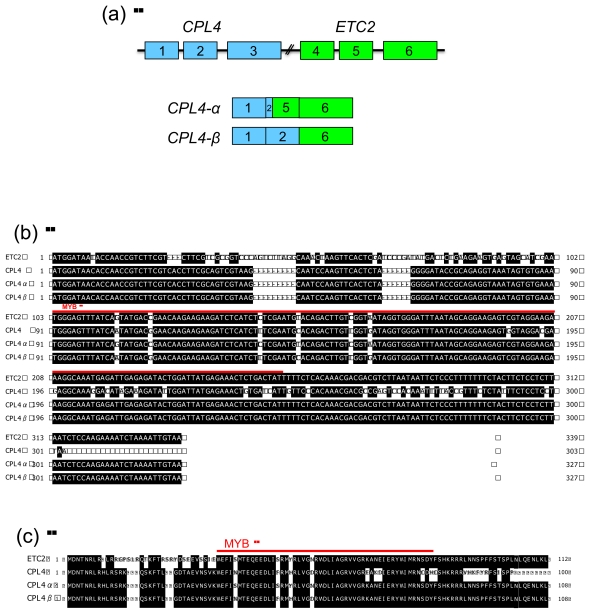
Gene structure and amino acid sequences of *CPL4*-*α* and *CPL4-β*. (**a**) Structure of *CPL4* and *ETC2* genes. The blue boxes 1, 2 and 3 represent *CPL4* exons. The green boxes 4, 5 and 6 represent *ETC2* exons. The black lines represent introns; (**b**) cDNA sequence alignment of *ETC2*, *CPL4*, *CPL4*-*α* and *CPL4-β* transcripts. Identical nucleotides are shaded in black. The red line indicates the MYB region; (**c**) Amino acid sequence alignment of ETC2, CPL4 and deduced CPL4-α and CPL4-β proteins. Identical amino acids are shaded in black. The red line indicates the MYB region.

To determine the precise structure of *CPL4*-related chimeric transcripts (Chimera 1 and 2), we cloned these RT-PCR products and subsequently sequenced the constructs. Chimera 1 included two different amplicons, named Chimera 1-1 and Chimera 1-2 (Figures 4c and 5b). Chimera 2 also included two different amplicons, named Chimera 2-1 and Chimera 2-2 (Figures 4c and 5b). As shown in Figure 5a, *CPL4* and *ETC2* lie in a tandem orientation (Figure 5a). All chimeric transcripts may result from alternative splicing of *CPL4* and *ETC2.* Chimera 1-1 and Chimera 1-2 contained the first two exons of *CPL4* and all three exons of *ETC2* (1, 2, 4, 5 and 6 exons) (Figure 5b). Both chimeric transcripts (Chimera 1-1 and Chimera 1-2) contain a TAGTT additional sequence between the second exon of *CPL4* and the first exon of *ETC2*. This linker sequence ‘TAGTT’ was just upstream of the 5′-UTR region of the first exon of *ETC2*. Although Chimera 1-1 included the first intron of *ETC2*, Chimera 1-2 did not (Figure 5b). Chimera 2-1 contained the first and second exons of *CPL4* and the third exon of *ETC2* (1, 2 and 6 exons) (Figure 5b). The “TAGTT” linker insertion of Chimera 1-1 and 1-2 was produced by GT-AG splicing between the second intron of *CPL4* and an upstream region of the first exon of *ETC2*. This linker sequence included a “TAG” sequence that could function as a stop codon. Therefore, Chimera 1-1 and 1-2 are expected to produce a truncated MYB protein that includes only the first two exons of *CPL4*. On the other hand, transcripts of Chimera 2-1 and 2-2 are expected to encode complete CPC-like MYB proteins. Chimera 2-1 contained the first two exons of *CPL4* and the third exon of *ETC2* (exons 1, 2 and 6) (Figure 5b). Chimera 2-2 contained the first *CPL4* exon, the first 16 bp of the second exon of *CPL4*, the second exon of *ETC2* lacking the first 16 bp, and the third exon of *ETC2* (exons 1, 2, 5 and 6) (Figure 5b). Thus, we renamed Chimera 2-1 and 2-2 to *CPL4-α* and *CPL4-β*, respectively (Figure 6a). *CPL4-*α shares 90% identity with *ETC2* and 83*%* identity with *CPL4* at the nucleotide level. *CPL4-β* shares 89% identity with *ETC2* and 84*%* identity with *CPL4* at the nucleotide level (Figure 6b). *CPL4-α* shares 86% identity with *ETC2* and 76*%* identity with *CPL4* at the amino acid level. *CPL4-β* shares 82% identity with *ETC2* and 80*%* identity with *CPL4* at the amino acid level (Figure 6c). Both CPL4-α and CPL4-β contain the conserved amino acid signature [D/E]Lx2[R/K]x3Lx6Lx3R that is required for the interaction with R/B-like bHLH transcription factors [39]. Their results strongly suggest that CPL4-α and CPL4-β serve as CPC-like MYB proteins harboring similar functions of ETC2 and/or CPL4.

Our study raises the possibility that there are unexpected alternative splicing sites spanning two homologous genes. In *Arabidopsis*, there are many examples of genes where alternative splicing provides a regulatory mechanism controlling aspects of development and other processes including flowering [40]. Although many genes produce alternatively spliced transcripts, the critical role of alternative splicing is poorly understood. This work provides additional evidence for the function of alternative splicing.

## 3. Experimental Section

### 3.1. Plant Materials and Growth Conditions

*Arabidopsis thaliana* ecotype Columbia (Col-0) was used as the wild type in this study. Seeds were surface-sterilized and sown on 1.5% agar plates as described previously [41]. Construction of the *35S::CPL3* transgenic line was described previously [31]. Seeded plants were kept at 4 °C for 2 days and then incubated at 22 °C under constant white light (50–100 μmol·m^−2^·s^−1^).

### 3.2. Gene Constructs

Sequences of all primers used in this paper are listed in Table 1. All PCR-generated constructs were completely sequenced following isolation of the clones to check for amplification-induced errors.

#### 3.2.1. *Promoter::GUS* Constructs

A 2.7 kb PCR-amplified promoter region of *CPL4* (primers NEKO45/NEKO47) was digested with *Sal*I and *Bam*HI and subcloned into pBluescript SK+ (Stratagene, La Jolla, CA, USA) to create *pBS-CPL4p*. The *Sal*I and *Bam*HI digested fragment of *pBS-CPL4p* was ligated into the *Sal*I and *Bam*HI sites of binary vector *pBI101* (Clontech Laboratories, Inc., Mountair View, CA, USA) to create the *CPL4p::GUS* constructs.

#### 3.2.2. *35S::ETC1* and *35S::ETC2* Constructs

A 0.5 kb PCR-amplified linear *ETC1* genome fragment (primers TW1169/TW1170), and a 1.0 kb PCR-amplified linear ETC2 genome fragment (primers TW1165/TW1166) were subcloned into *pBS* using Pyrobest DNA polymerase (Takara, Tokyo, Japan) to make *pBS-ETC1* and *pBS-ETC2*. Next, *Acc65I* to *Sal*I fragments were ligated into the *Acc65I* to *Sal*I sites of the *pCHF3* binary vector [42] to create *35S::ETC1* and *35S::ETC2*.

### 3.3. Transgenic Plants

Plant transformation was performed by a floral dip method [43], and transformants were selected on 0.5 × MS agar plates containing 50 mg/L kanamycin. Homozygous transgenic lines were selected for kanamycin resistance. At least twelve T1 lines were isolated for each construct and at least six T2 and three T3 lines were selected on the basis of their segregation ratios for kanamycin resistance.

### 3.4. RNA Isolation and Semi-Quantitative RT-PCR

Total RNA was extracted using RNeasy Plant Mini Kits (Qiagen, Valencia, CA, USA). On-column DNase I digestion was performed during RNA purification following the protocol described in the RNeasy Mini Kit handbook. First-strand cDNA was synthesized from 1 μg total RNA in a 20 μL reaction mixture using the Prime Script RT Regent Kit (Takara).

Semi-quantitative RT-PCR reactions were conducted as described by Kurata *et al.* [44]. The *CPL4* fragment was amplified with RT341/RT342 primer pairs. The *CPC* fragment was amplified with RT128/RT129 primer pairs. The *ETC2* fragment was amplified with RT124/RT125 primer pairs. *EF* was amplified with the EF1α-F/EF1α-R primer pair as described by Kurata *et al.* [21]. To determine the sequence of the PCR products, we cloned them into the pT7 blue T-vector (Novagen) and subsequently sequenced the constructs.

### 3.5. Histology

*Promoter::GUS* plants were excised from the growth medium and immersed in X-Gluc solution containing 1.0 mM X-Gluc (5-bromo-4-chloro-3-indolyl-β-glucuronide), 1.0 mM K_3_Fe(CN)_6_, 1.0 mM K_4_Fe(CN)_6_, 100 mM NaPi (pH 7.0), 100 mM EDTA and 0.1% Triton X-100. Primary roots of 7-day- or two-week-old seedlings were incubated at 37 °C overnight. Cotyledons of 7-day-old seedlings and two-week-old rosette leaves were incubated at 37 °C for 3 h.

### 3.6. Microscopy

Root GUS activity was observed using an Olympus Provis AX70 microscope. At least five individual primary roots of 7-day- and two-week-old seedlings were analyzed for root GUS activity. To observe leaf GUS activity, an Olympus SZH binocular microscope was used. At least five cotyledons of 7-day-old seedlings and two-week-old rosette leaves were analyzed for leaf GUS activity.

### 3.7. Accession Numbers

CPC (At2g46410), TRY (At5g53200), ETC1 (At1g01380), ETC2 (At2g30420), CPL3/ETC3 (At4g01060), CPL4 (At2g30424) and TCL1 (At2g30432).

## 4. Conclusions

In this paper, we investigated the expression of *CPL4* and *CPL4*-related chimeric transcripts in *Arabidopsis* using promoter-GUS and RT-PCR analyses. The results showed that *CPL4* is specifically expressed in leaf blades, and *CPL4* expression was not affected by *ETC1*, *ETC2* or *CPL3* overexpression. We hypothesize that the proteins created by inter-genic alternative splicing between *CPL4* and *ETC2* act in a redundant manner with other CPC-like MYB family proteins. Much work remains to determine the function of *CPC*-like MYB gene family members. By overexpressing 35S::*CPL4-α* and 35S::*CPL4-β* and investigating the localization of CPL4::CPL4-α:GFP and CPL4::CPL4-β:GFP in transgenic plants, we should be in a better position to elucidate the roles of these *CPC*-like MYB genes in *Arabidopsis* epidermal cell differentiation.

## Figures and Tables

**Table 1:** Primer sequences used in this study.

Primer Name	Sequence
RT124	5′-GATAATACCAACCGTCTTCGTCTTC-3′
RT125	5′-TTCTTGGAGATTAAGAGGAGAAGTAG-3′
RT128	5′-CTTCTTGTTTCTCGAGATTTATTCTC-3′
RT129	5′-AATAGTAATTCAAGGACAGGTACATTTC-3′
RT318	5′-GAATTATTAAGACGTCGTCGTTTGTGAG-3′
RT341	5′-AAGCAATCCAAGTTCACTCTAGGG-3′
RT342	5′-CGGTAAATTTGTGGACTCGG-3′
NEKO45	5′-ATATGTCGACTACCAAAATCACTCCACCATTTTC-3′
NEKO47	5′-ATATGGATCCGTTGGTGTTATCCATTGGTATTTG-3′
TW1165	5′-ATATGGTACCAATAAAAAATAAATCAC-3′
TW1166	5′-TGCTTGTCGACTGTATACACTAA-3′
TW1169	5′-ATATGGTACCACTTCATGTTCTTCCCTT-3′
TW1170	5′-ATATGTCGACAAGCCAATACATATCCA-3′
